# Evaporation kinetics in highly porous tetrapodal zinc oxide networks studied using in situ SRµCT

**DOI:** 10.1038/s41598-021-99624-y

**Published:** 2021-10-12

**Authors:** Birte Hindenlang, Anna Gapeeva, Martina J. Baum, Sören Kaps, Lena M. Saure, Florian Rasch, Jörg Hammel, Julian Moosmann, Malte Storm, Rainer Adelung, Fabian Schütt, Berit Zeller-Plumhoff

**Affiliations:** 1 Institute of Metallic Biomaterials, Helmholtz Zentrum Hereon GmbH, Max-Planck-Straße 1, 21502 Geesthacht, Germany; 2 Functional Nanomaterials, Institute for Materials Science, Kiel University, Kaiserstr. 2, 24143 Kiel, Germany; 3 Institute of Materials Physics, Helmholtz Zentrum Hereon GmbH, Max-Planck-Straße 1, 21502 Geesthacht, Germany; 4 Diamond Light Source Ltd., Diamond House, Harwell Science and Innovation Campus, Didcot, OX11 0DE UK

**Keywords:** Ceramics, Materials science, Imaging techniques

## Abstract

Tetrapodal zinc oxide (t-ZnO) is used to fabricate polymer composites for many different applications ranging from biomedicine to electronics. In recent times, macroscopic framework structures from t-ZnO have been used as a versatile sacrificial template for the synthesis of multi-scaled foam structures from different nanomaterials such as graphene, hexagonal boron nitride or gallium nitride. Many of these fabrication methods rely on wet-chemical coating processes using nanomaterial dispersions, leading to a strong interest in the actual coating mechanism and factors influencing it. Depending on the type of medium (e.g. solvent) used, different results regarding the homogeneity of the nanomaterial coating can be achieved. In order to understand how a medium influences the coating behavior, the evaporation process of water and ethanol is investigated in this work using in situ synchrotron radiation-based micro computed tomography (SRµCT). By employing propagation-based phase contrast imaging, both the t-ZnO network and the medium can be visualized. Thus, the evaporation process can be monitored non-destructively in three dimensions. This investigation showed that using a polar medium such as water leads to uniform evaporation and, by that, a homogeneous coating of the entire network.

## Introduction

**Figure 1:**
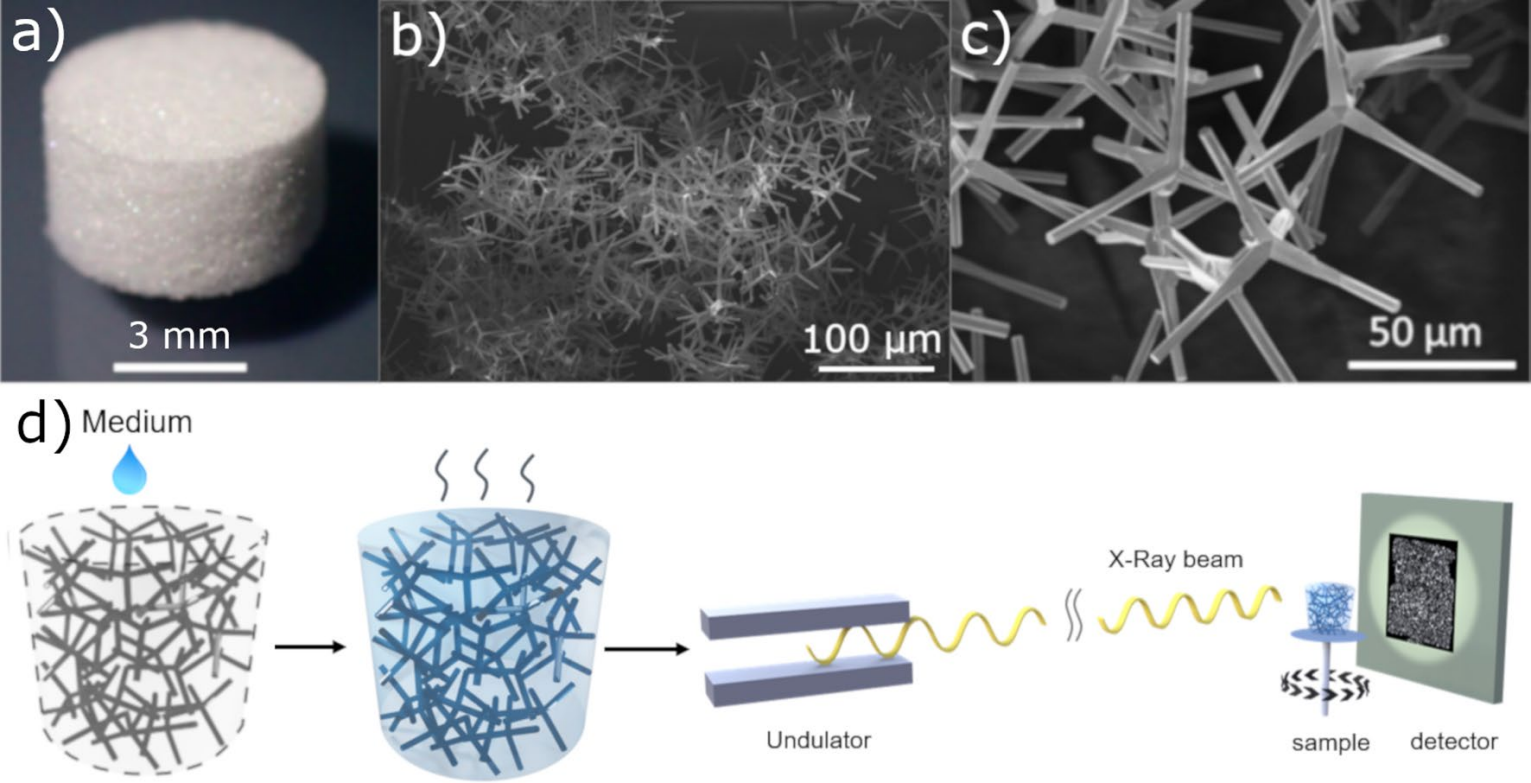
(**a**) Exemplary image of a fabricated t-ZnO template with a porosity ~ 94%, (**b**) low magnification SEM micrograph of t-ZnO powder, (**c**) high magnification SEM micrograph of t-ZnO powder produced by the FTS approach, and (**d**) schematic illustration of the experimental procedure. In the first step, the porous t-ZnO network is infiltrated with the medium. In the next step, the medium is evaporating in situ during the imaging of the process using SRµCT.

ZnO is a direct wide bandgap (~ 3.4 eV) ceramic n-type semiconductor that crystallizes preferentially in the hexagonal wurtzite-type structure and, depending on the synthesis route, exists in multiple nano- and microstructures, e.g., rods, belts, tubes, flowers, and tetrapods^1–3^. The unique 3D structure of tetrapods is characterized by four arms connected by a core at an angle of ~ 110° with respect to each other (Fig. 1b,c)^4^. Tetrapodal-shaped ZnO (t-ZnO) is one of the most multifunctional materials that can be used on its own or as a template for the fabrication of sophisticated nanocomposite and highly porous 3D materials^3^. Different strategies have been developed for the synthesis of tetrapodal-shaped particles, including vapor-phase growth, wet chemical and hydrothermal methods, microwave-assisted growth, DC thermal plasma and pulsed laser deposition, as well as flame transport synthesis (FTS)^3^. The latter provides a simple solvent-free method for the fabrication of t-ZnO microparticles on a large scale and enables defined control over the aspect ratio (length:diameter) and morphology of the t-ZnO arms^4,5^. When t-ZnO particles are molded and sintered, 3D networks with a large free volume as well as a large surface-to-volume ratio can be achieved, providing networks with high mechanical flexibility and enhanced sensing properties. The porosity of such structures can be tuned by the amount of t-ZnO used during fabrication and can be as high as 98%^3,6^. Moreover, the surface of t-ZnO particles can be coated by another material using chemical vapor deposition (CVD) techniques or by infiltration of nanoparticle dispersions and polymer solutions^7–9^. The t-ZnO can then be removed by wet-chemical etching using, for example, acetic or hydrochloric acid solutions. Removal of t-ZnO results in the formation of interconnected hollow tetrapod-like structures with wall thicknesses in the nanometer range^7,10–12^. Based on their multi-scaled nature, these framework structures show remarkable mechanical, electrical and optical properties, suitable for applications in the fields of sensing, photocatalysis and water purification, photonics, actuation, energy storage and biomedicine^3,4,13–19^.

Being simple, cost-effective, and at the same time a versatile technique, the solution-based infiltration approach provides several advantages over CVD and is, therefore, the predominant choice for the fabrication of 3D network structures based on t-ZnO^7^. Due to the high porosity of the hydrophilic t-ZnO network (pores in the micrometer range) and strong capillary forces, no self-filtering effect occurs even at a high concentration of nanoparticles, with the concentration being limited by the dispersion stability and not by the infiltration process^7,11^. However, in order to design 3D structures with controlled and predictable properties, it is crucial to understand the evaporation process of the dispersion medium/solvent. Up to now, this process could only be evaluated after the experiment by using SEM, i.e., after evaporation of the dispersion medium/solvent, based on the homogeneity of the nanoparticles or polymer distribution within the network. Generally, it was observed that the use of polar dispersion media such as water leads to the most uniform coverage of t-ZnO surfaces by nanoparticles. Furthermore, due to the hydrophilicity of t-ZnO, it is assumed that during the drying process, the water layer becomes thinner while forming menisci between the tetrapod arms and at interconnection points to minimize its surface area, which leads to sail-like formations^7,11^. Nevertheless, the evaporation process in general is not trivial but underlies several complicated mechanisms.

In this work, we are strengthening the understanding of the evaporation process inside the network by employing in situ synchrotron X-ray radiation-based micro computed tomography (SRµCT). This method enables the dynamic and non-destructive imaging of the whole sample while achieving a high spatial and temporal resolution. By this, the whole evaporation process inside the sample can be visualized from the beginning until the medium is fully evaporated. X-ray computed tomography utilizes the different attenuation lengths of materials to create a contrast between materials which can be detected^20^. For the visualization of low attenuating materials such as water, propagation-based phase contrast is required^21^.

As shown in Fig. 1d, cylindrical samples of t-ZnO networks are infiltrated with water or ethanol and afterwards imaged in situ using SRµCT. The analysis of these 4D datasets enables a better understanding of the evaporation and by that, the coating of the network.

## Materials and methods

### Fabrication of samples

Five cylindrical samples with a porous density of 0.3 g/cm^3^ were investigated. The t-ZnO powder was fabricated using the Flame Transport Synthesis (FTS), as described before in^4^. Afterwards, the powder was pressed into a cylindrical shape (see Fig. 1a–c) with the dimensions of 3 mm height and 3 mm diameter. For more stability and to form connections between the tetrapods, the samples were sintered in an oven (Nabertherm B180) for 5 h at 1150 °C.

### SRµCT imaging of samples

The prepared samples were imaged in situ using SRµCT at the Diamond Manchester Imaging Branchline I13-2 at Diamond Light Source (Didcot, UK)^22^ with a pink beam with a mean energy of 26 keV and a 500 µm thick LuAG scintillator and a pco.Edge 5.5 sCMOS camera. To enable propagation-based phase contrast, the sample-to-detector distance was set to approx. 50 mm. In total, five samples were investigated slightly above room temperature, see also Table 1. For heating, a Deben Rig DB5000 heating plate was used. Each sample was infiltrated with 20 µl water or ethanol and afterwards imaged. The tomographic imaging was continued until no more medium was visible. Some time passed between infiltration and imaging because they were done in different rooms. To measure the temperature for all samples, a sensor was placed 5 mm above the sample. Four samples were infiltrated with water and one with ethanol. Additionally, one water infiltrated sample was imaged at 8× magnification and one with a reduced exposure time. These samples should help to investigate the evaporation directly at the tetrapods but also the influence of the imaging. The results of these samples are given in the Supplementary Data.

**Table 1:** Information of the imaging conditions for each sample.

Sample	Temperature [°C]	Rel. humidity (end of imaging) [%]	Magnification	Voxel Size [µm]	Exposure time [ms]	Scan time [min]	Medium
1	29.5	55.4	4x	1.6	150	152	Water
2	24.0	60	4x	1.6	150	216	Water
3	21.7	60.4	8x	0.8	150	64	Water
4	21.5	63	4x	1.6	50	72	Water
5	21.7	58	4x	1.6	150	18	Ethanol

All samples were scanned continuously in fly-scan mode (continuous rotation around 360°) with 2160 projections per tomogram, including 40 dark and flat field images at the beginning and 40 flat field images at the end. The phase retrieval and the reconstruction were performed using the open-source Savu framework^23^ and the TomoPy reconstruction package^24^. Projections were corrected for ring artefacts^25^. For the Paganin phase retrieval, a δ-to-β ratio of 50 was found to yield the images with the highest contrast and little blurring of the fine t-ZnO structures. Sample 4 was imaged using a shorter exposure time to enable a more close investigation of the evaporation process.

Due to the high evaporation rate of ethanol, no relevant information could be gained for the analysis of the evaporation process. The time series of the ethanol evaporation process are shown in the Supplementary Data Figure A1.

## Results and discussion

**Figure 2:**
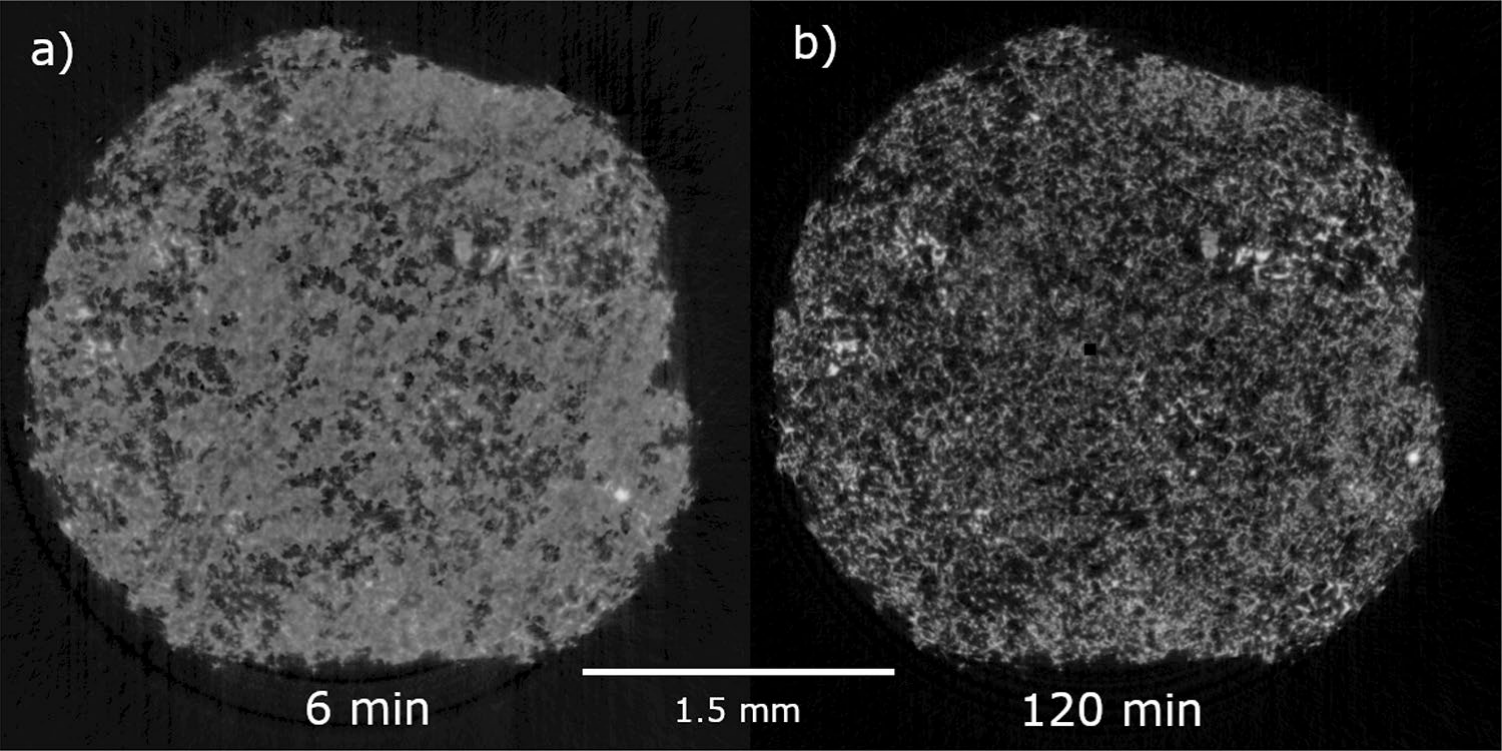
Tomograms of the t-ZnO network infiltrated with water after 6 min (**a**) and 120 min (**b**) imaging time. The water in (**a**) is clearly visible compared to the dry t-ZnO network in (**b**). In the beginning, large pores without water are already visible.

The tomograms, as shown in Fig. 2, were analyzed as described before. In these exemplary tomograms, the difference between the infiltrated sample (a) and the dry t-ZnO network (b) is clearly visible due to the phase-contrast imaging.

### t-ZnO arm and pore size distribution

**Figure 3:**
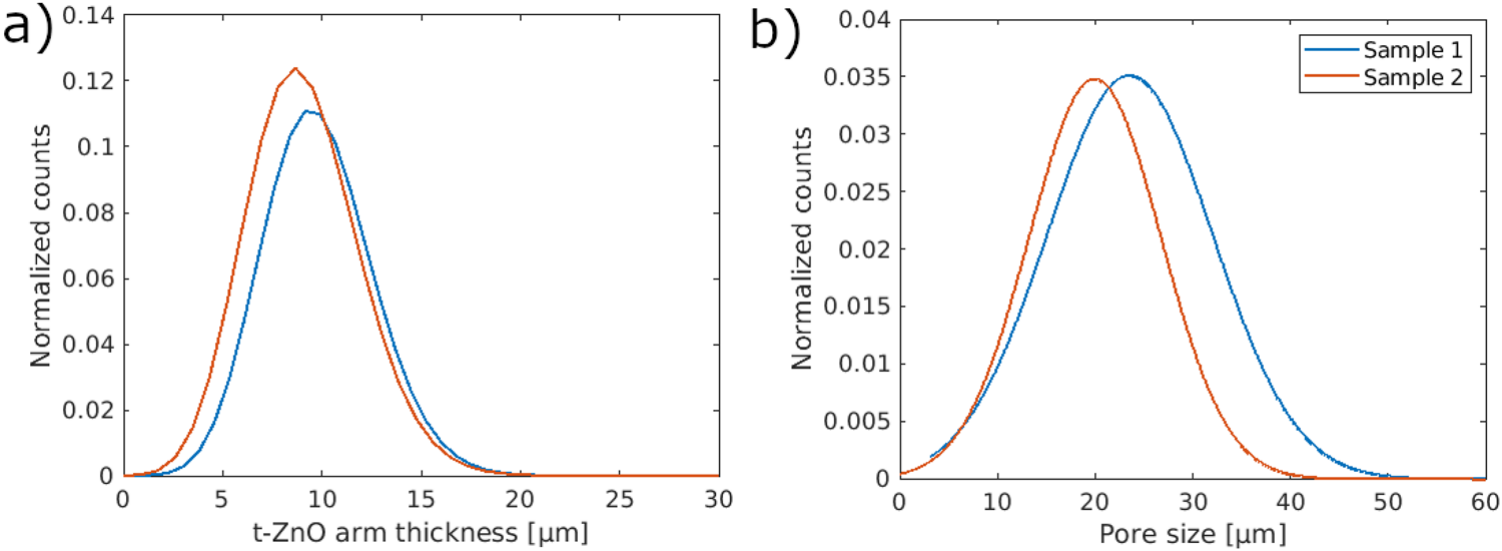
The t-ZnO arm thickness distributions in (**a**) show that sample 2 has slightly smaller thicknesses as sample 1. A similar trend is observed for the pore size distributions (**b**). Here, sample 2 indicates slightly smaller pores than sample 1.

The t-ZnO arm thickness distributions (Fig. 3a) of the samples 1 and 2 are very similar with just small variations. For these two samples, the calculated mean values are in the same range with 9.82 µm and 9.07 µm, respectively. Structures larger than 15 µm are seldomly detected, while below 3 µm no structures are resolved (see also Supplementary Data Figure A5). Both the t-ZnO arm thickness and the pore size distribution were calculated by fitting spheres into the structures.

One reason why no structures smaller than 3 µm are detected is the registration process along with the resolution limit of the detector. The effective voxel size of the detector system was 1.6 µm, meaning that structures smaller than this size cannot be detected. Structures with the size of one voxel were deleted during the segmentation and registration process so that only structures above 3 µm remain. In the literature, mean arm thicknesses ranging from 3 µm to 5 µm are reported^4^. These values are only reached for the 8× magnification in this work (see Supplementary Data Figure A5). This suggests that the more accurate estimation of the tetrapod arm thickness is achieved using a higher magnification. Another important factor regarding the different magnifications is the partial-volume effect. This is reduced for higher magnifications leading to more accurate results. Independent of the magnification, the propagation-based phase contrast leads to larger values for tetrapods^26,27^.

The pore size distributions (Fig. 3b) show similar trends as the t-ZnO arm thickness distribution with slightly larger differences between the mean values. The samples 1 and 2 have mean pore sizes of 24.98 µm and 20.69 µm, respectively. The range of possible pore sizes is much broader than for the t-ZnO arm thicknesses. However, nearly no pores are detected with sizes smaller than 10 µm and there are slightly more smaller-sized pores than larger ones which can be seen in the counts in the individual distributions (see Supplementary Data Figure A5).

### Overall water and t-ZnO content

**Figure 4:**
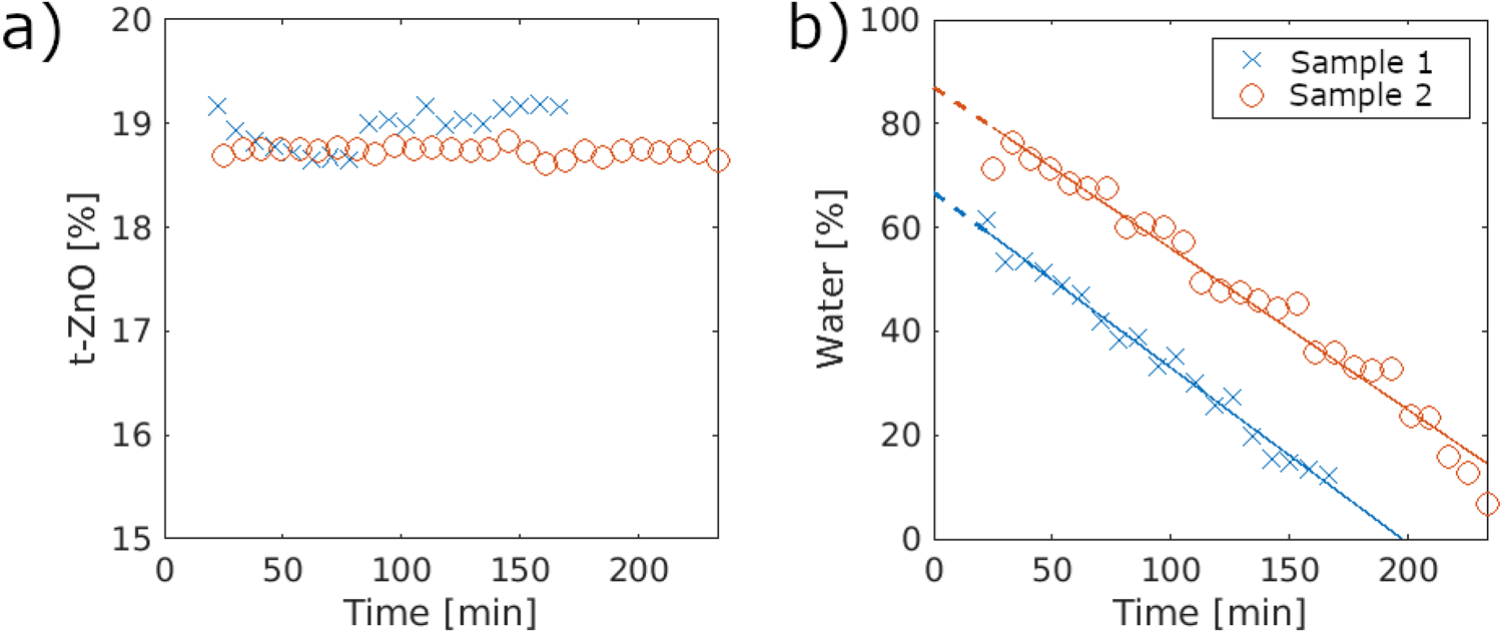
The t-ZnO distribution in (**a**) shows that for sample 2 a nearly ideal, constant distribution over time is observed while sample 1 has slight changes over time. For the water distributions in (**b**), linear evaporation behaviors are seen. In comparison, both curves seem identical but shifted.

For both water and t-ZnO content, a grey-value segmentation was done. By this, the calculated content values are volume percentages rather than weight percentage. The overall t-ZnO contents for all samples in Fig. 4a show that the general content is around 19%. Both samples show only small deviations in the t-ZnO content whereas sample 2 shows a nearly ideal behavior over time with no significant changes. Small deviations of sample 1 are an example of the influence of the segmentation where the intra-observer variability or the registration process affects the resulting t-ZnO content^28,29^.

Both samples show that the water content decreases strongly with a linear trend (see Fig. 4b). Small increases in water content of approx. 1% from one time step to the next can be seen especially for sample 2 but also for sample 1. Due to the intra-observer variability, this error is neglectable. The data points in Fig. 4 do not start right away since some time passed between the infiltration and the imaging start. For the water content in Fig. 4b, the linear regression is extrapolated to indicate the previous evaporation. The initial water content estimated by the extrapolation should be similar in both cases.

The resulting evaporation rates along with the initial water content and the R^2^ value as an indication of the quality of the fit are given in Table 2.

**Table 2:** Fitting parameters and errors of the linear fits of the form f (x) = ax + b of the water evaporation.

	Sample 1	Sample 2
Evaporation rate a [%/min]	− 0.34	− 0.31
Initial water content b [%]	62.01	81.72
Quality of fit R^2^	0.98	0.97

Comparing the evaporation rates demonstrates that sample 1 and sample 2 have nearly identical rates of 0.34%/min and 0.31%/min, respectively. In both cases, the R^2^ value is above 0.97, which indicates a good fit.

In theory, a higher temperature should lead to faster water evaporation so that the evaporation rate of sample 1 should be the highest^30^. Since the temperature difference between sample 1 and 2 is only 5.5 °C this effect can be neglected. Additionally, the evaporation rates are nearly identical which indicates that the small differences between the t-ZnO networks of both samples have no influence. The only major difference between the samples is the time shift of the evaporation seen in Fig. 4b. Since some time passed between infiltration and imaging, indicated by the extrapolation in Fig. 4b, the initial evaporation behavior is unknown. The extrapolation suggests that a faster evaporation is possible in the beginning. Otherwise both samples would not have been completely infiltrated by water. This difference in water content in the beginning of the imaging may be due to the lower humidity of air for sample 1. For a lower humidity, a faster evaporation is possible.

### Radial and height distribution of water and t-ZnO

**Figure 5:**
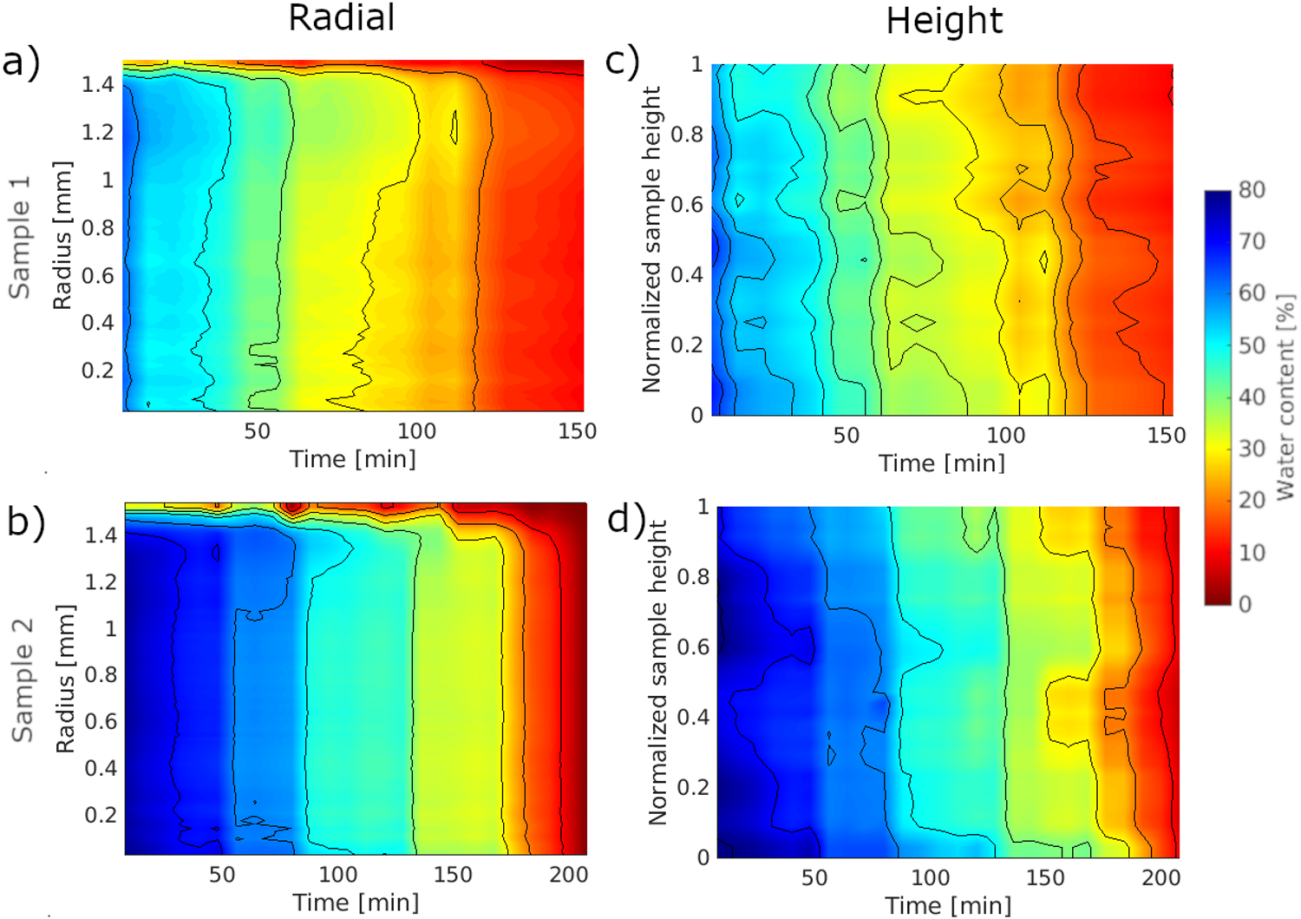
Radial (left column) and height (right column) distributions of water content in (**a**),(**c**) sample 1, (**b**),(**d**) sample 2 (created with MATLAB). For both samples, the water content is nearly equal over all radii except the outermost regions. This effect enhances over time until roughly 10% of water remains. Similarly, the water content is uniform over the whole height of the sample with slightly more water at the bottom.

The radial distributions of water given in Fig. 5a,b show a continuous decrease in water content. Regardless of the sample, the water content at a fixed time point is almost constant across all radii with lower contents at the outermost radii. These regions of lower water content grow until approximately 10% of water remains. In the case of the radial distribution of the t-ZnO content (see Supplementary Data Figure A4), no significant changes can be seen over time for sample 1. In general, the t-ZnO content is equal across all radii except the outermost, where a lower content is observed. This is expected since the outer shapes of the samples are not smooth, and by that, lower contents are expected.

Unlike the radial distribution, the height distribution of water (Fig. 5c,d) shows a less homogeneous distribution over the height of the sample. The water content is usually higher at the bottom of the sample and small but significant variations over the height of the sample are seen. Additionally, the evaporation is slower at the bottom of the sample for two samples. Since the sample is placed on a sample holder, no water can evaporate in this direction as opposed to the open top. In contrast to the water content, the t-ZnO content is rather stable over the height of the sample with a slightly higher amount of t-ZnO at the bottom (see Supplementary Data Figure A4). Over time, the t-ZnO content stays relatively constant except for sample 1 after roughly 120 min. Here, the t-ZnO content is very low apart from the upper half of the sample. This drop in t-ZnO content is also visible in the overall t-ZnO content in Fig. 4 and may be due to uncertainties in the segmentation.

### Model of water evaporation behavior

To propose a possible model of the evaporation process, the sample (sample 3) imaged with 8× total magnification (corresponding to an effective pixel size of 812 nm) is analyzed.

**Figure 6:**
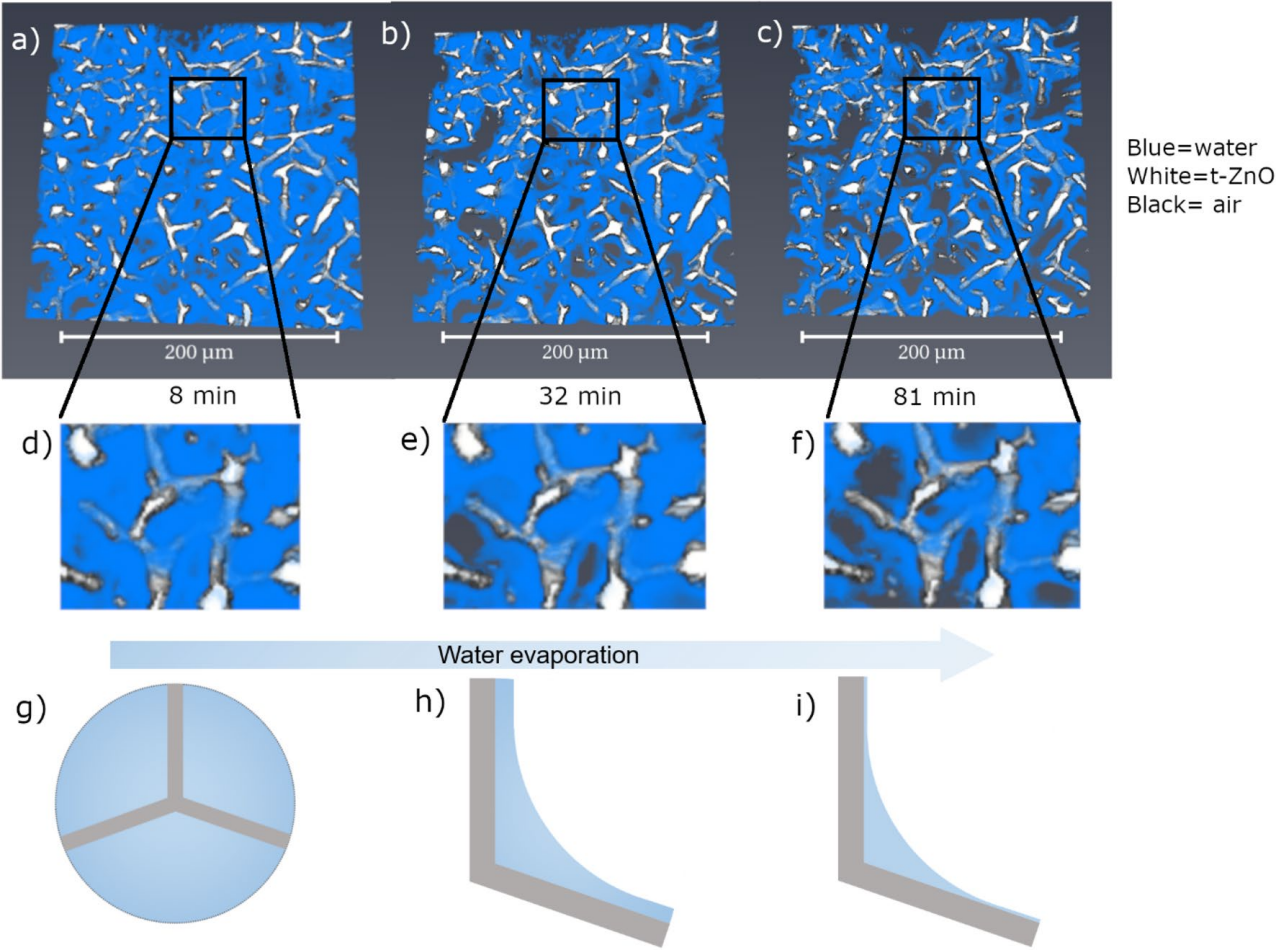
Evaporation process of water of sample 3 in the t-ZnO network (**a**–**c**). At first (**a**), nearly the whole sample is wetted by the medium, with only very large pores being without the medium. As seen in (**d**) and schematically in (**g**), the tetrapods are completely surrounded by the medium. After some time (**b**) the areas without medium grow so that menisci form around the tetrapod arms. This is shown in (**e**) and schematically in (**h**), where the water layer is reduced, but the whole tetrapod is still surrounded by the medium. With further evaporation (**c**), smaller pores can be seen without the medium. The tetrapod arms are now surrounded by only a thin layer of medium (**f**) while there is still a meniscus, as shown in (**i**).

Overall, the water evaporation showed several specific trends which can be summarized to obtain a model of the evaporation inside the t-ZnO network. In general, a continuous linear decrease in water content is seen, which would be expected for a single air–water interface rather than for multiple interfaces, as in the case of a t-ZnO network^30^. This implies that regardless of the many air–water interfaces, the evaporation still behaves macroscopically as a single air–water interface. To evaluate the microscopic evaporation behavior, one has to look at the evaporation behavior at one tetrapod, as shown in Fig. 6. Figure. 6 shows a small region of sample 3 (RT, 8× magn.) at the starting point, an intermediate time point, and nearly at the end of the imaging process. At the beginning of the imaging, the sample is nearly completely wetted by water (Fig. 6a), while the pores are less filled with water for the later time points (Fig. 6b,c). As can be seen in Fig. 6d–f, the large pores display a faster reduction in water content, while the water remains close to the t-ZnO structures. The process around the tetrapod is schematically shown in Fig. 6g–i. This evaporation process is reasonable since t-ZnO is hydrophilic and it shows that the water layer around the tetrapods is thinning over time so that the tetrapod remains covered until the sample is dry. This effect was also observed in other studies^11,31^. Even though the ethanol evaporation was too fast for an accurate evaluation, a very similar trend can be seen (see Supplementary Data Figure A1). We did not consider other commonly used non-polar solvents such as hexane or pentane since we expect them to evaporate even more quickly than ethanol due to their lower boiling points.

**Figure 7:**
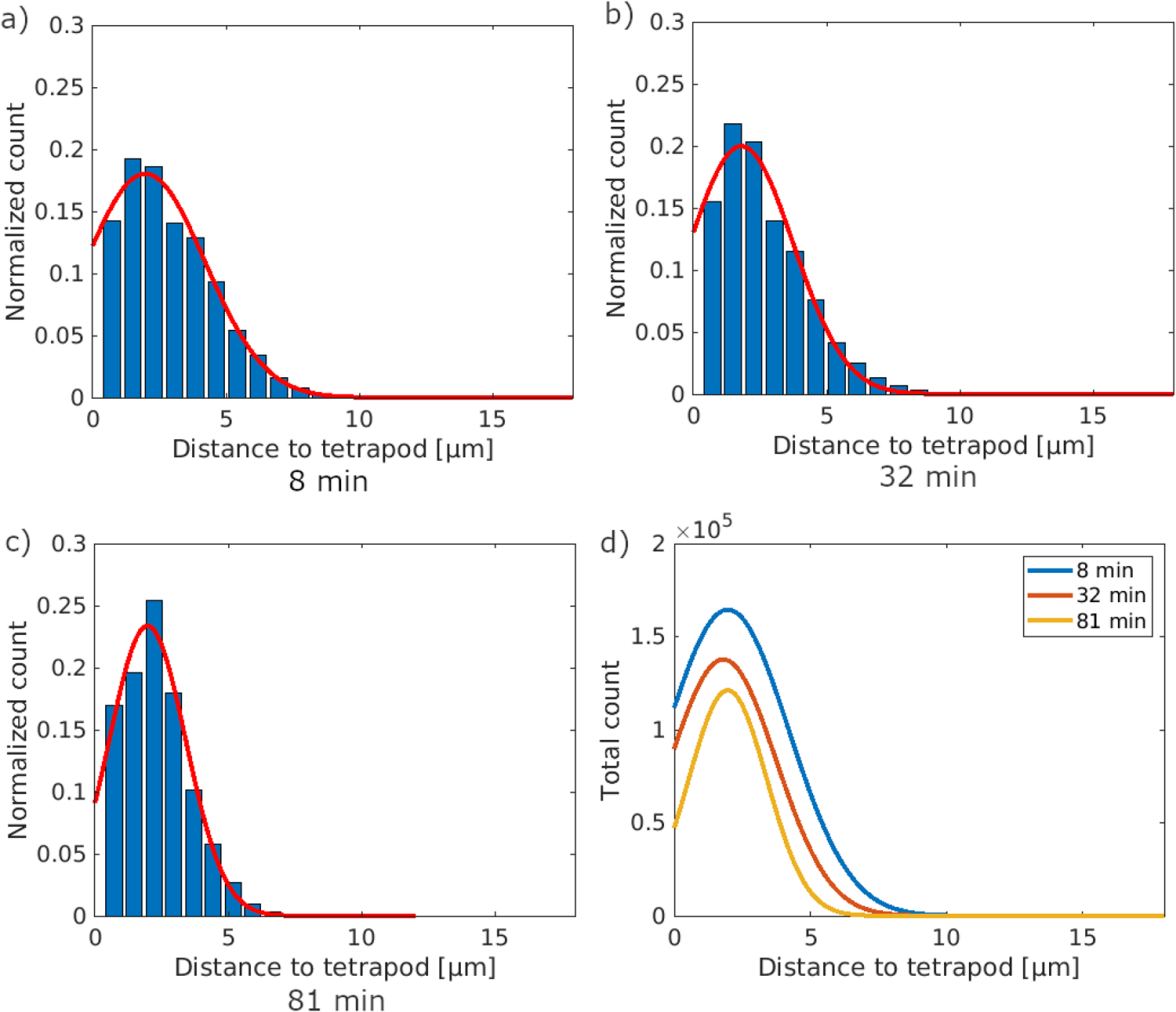
Distance distribution of water to the tetrapod arms of the three areas shown before. In orange, the Gaussian fit of the distribution is indicated. Overall, the distributions seem similar, while at the beginning (**a**), the occurring distances are distributed broader. Over time, the distribution narrows down to smaller distances (**b**,**c**) both for the measured maximum distances but also for the maximum peaks. Comparing all three distributions and the total counts (**d**), it is shown that the highest and broadest distribution of water distances is reached in the beginning.

Since the water evaporates faster in larger pores, samples with large pores should exhibit higher evaporation rates. Figure 7 shows histograms of the distance of the individual water voxel to the tetrapod arms for the three sections given in Fig. 6. For each voxel containing water, the minimum distance to a tetrapod arm was calculated. Comparing the mean distances of 2.9 µm, 2.6 µm, and 2.3 µm for 8 min, 32 min, and 81 min, respectively, a small reduction in distance is observed. This trend is confirmed by the decreasing detection of large distances while the amount of small distances increases which can be seen in Fig. 7d. In theory, the difference between the mean distances of all three time points should be more pronounced than calculated since the water layer thins more strongly than shown in the graphs. The actual small difference is due to the high number of water containing voxels at smaller distances which is also considered for the mean value calculation. This leads to a stronger weight for lower values and overall smaller distances. Nevertheless, this shows that over time, the water layer around the tetrapod arms is thinning while directly at the tetrapods the water remains in form of menisci, as shown in Fig. 6.

The microscopic evaporation process alone would suggest that with lower water contents, the air–water interfaces would grow, which would lead to an increase in the evaporation rate. As was observed before in case of water drops, the radius of the drop is determining the evaporation rather than the surface area^32^. The effect that the evaporation rate increases probably does not occur because the evaporation inside the sample leads to the saturation of the enclosed air and relatively equal evaporation occurs. At the outer edges of the sample, the air convection from the air conditioning and natural diffusion of the humidity lead to a lower partial water pressure and allow constant evaporation^32^. The obtained results are in accordance with publications, where aqueous nanoparticle dispersions, e.g., CNT dispersions, were used and homogeneous coverage of the t-ZnO networks due to homogeneous evaporation of the dispersion medium was observed^7,11^.

## Conclusion

The investigation using propagation-based phase contrast SRµCT enabled the visualization of the dynamic evaporation process of water from t-ZnO networks for the first time. It was shown that the evaporation from a t-ZnO network is homogeneous across the whole sample. Evaporation rates were not influenced by small variations in the t-ZnO arm thickness and pore size of the samples.  This allows for a homogeneous coating of the t-ZnO network using water as a dispersion medium, which agrees with previous observations regarding polar media^7^. Even though the evaporation rate of ethanol was too high for a detailed evaluation, the obtained results are promising regarding the proposed evaporation model. Furthermore, this study helps in understanding the evaporation process in other porous media.

## Supplementary Information

Supplementary Information.
