# Supplementary material for: Evaporation kinetics in highly porous tetrapodal zinc oxide networks studied using in situ SRµCT
Source: Sci Rep. 2021 Oct 12;11:20272. doi: 10.1038/s41598-021-99624-y (PMC8511110; doi:10.1038/s41598-021-99624-y)
Supplement: Supplementary file 1 — Supplementary Information. [file 41598_2021_99624_MOESM1_ESM.pdf]

Supplementary Data

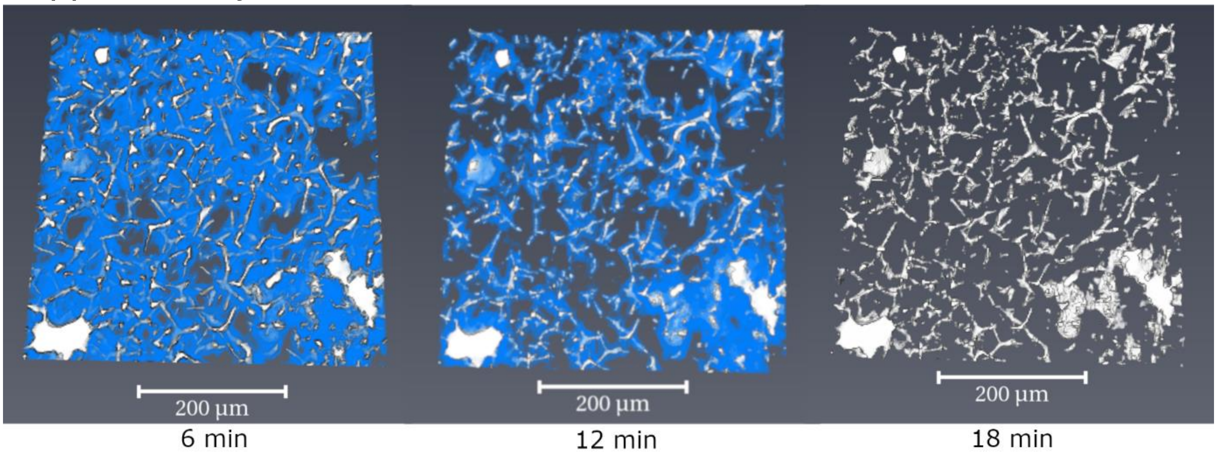

Figure A1: The evaporation process of ethanol after 6, 12, and 18 min. Since the evaporation process was very fast and after 18 min, no ethanol remained in the t-ZnO network, a representative analysis of this process was not possible. Nevertheless, the trend seen for the water evaporation is also visible for the ethanol.

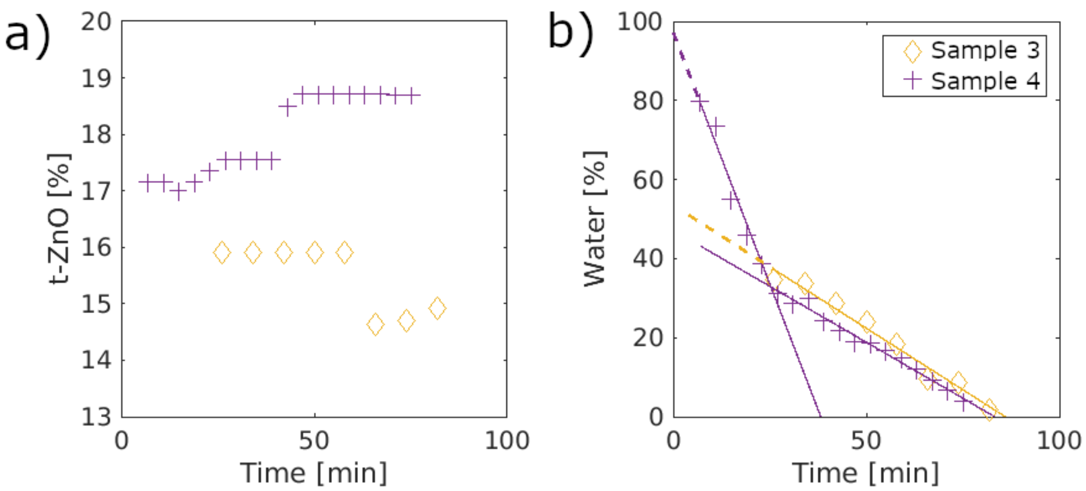

Figure A2: Overall t-ZnO content (a) and water content (b) for the samples 3 and 4 over time. In both cases, the t-ZnO content changes rather strongly over time which is due to the segmentation process. For sample 3 which was imaged with 8x magnification, a lower t-ZnO content is detected due to the higher magnification. The water content decreases similarly while for sample 3 a faster evaporation is seen in the first approx. 20 min. This enhances the suggestion that a different evaporation mechanism dominates in the beginning.

Table A1: Fitting parameters and errors of the linear fits of the form f (x) = ax+b of the water evaporation for sample 3 (8x magnification) and sample 4 (shorter exposure time)

	Sample 3	Sample 4
Evaporation rate<br>a [%/min]	-0.62	-2.54<br>-0.57
Initial water content<br>b [%]	42.3	89.59<br>45.46
Quality of fit<br>R^2	0.97	0.97<br>0.98

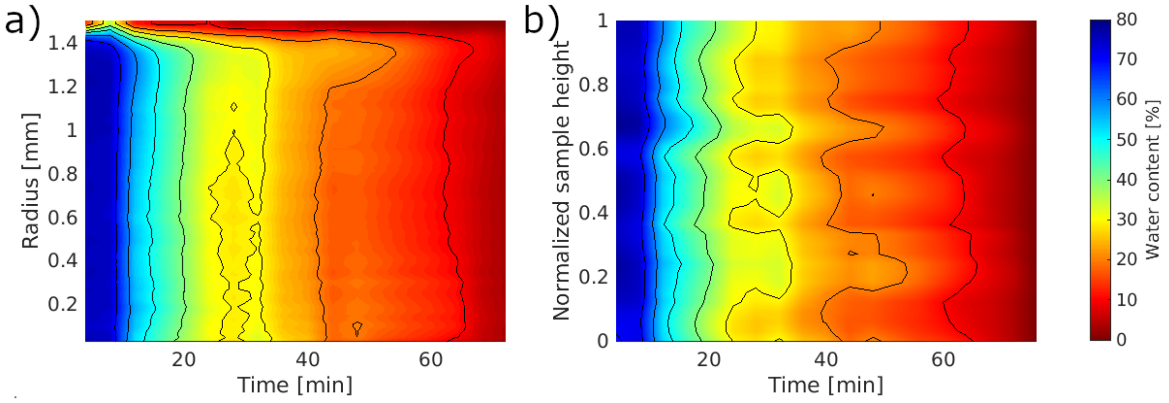

Figure A3: Shown is the radial distribution (a) and height distribution (b) of water in sample 4, which has a shorter exposure time. Similar to the distributions of sample 1 and 2 a rather constant water content is observed over all radii except the outermost regions. Here, the water content is lower than in the remaining sample.

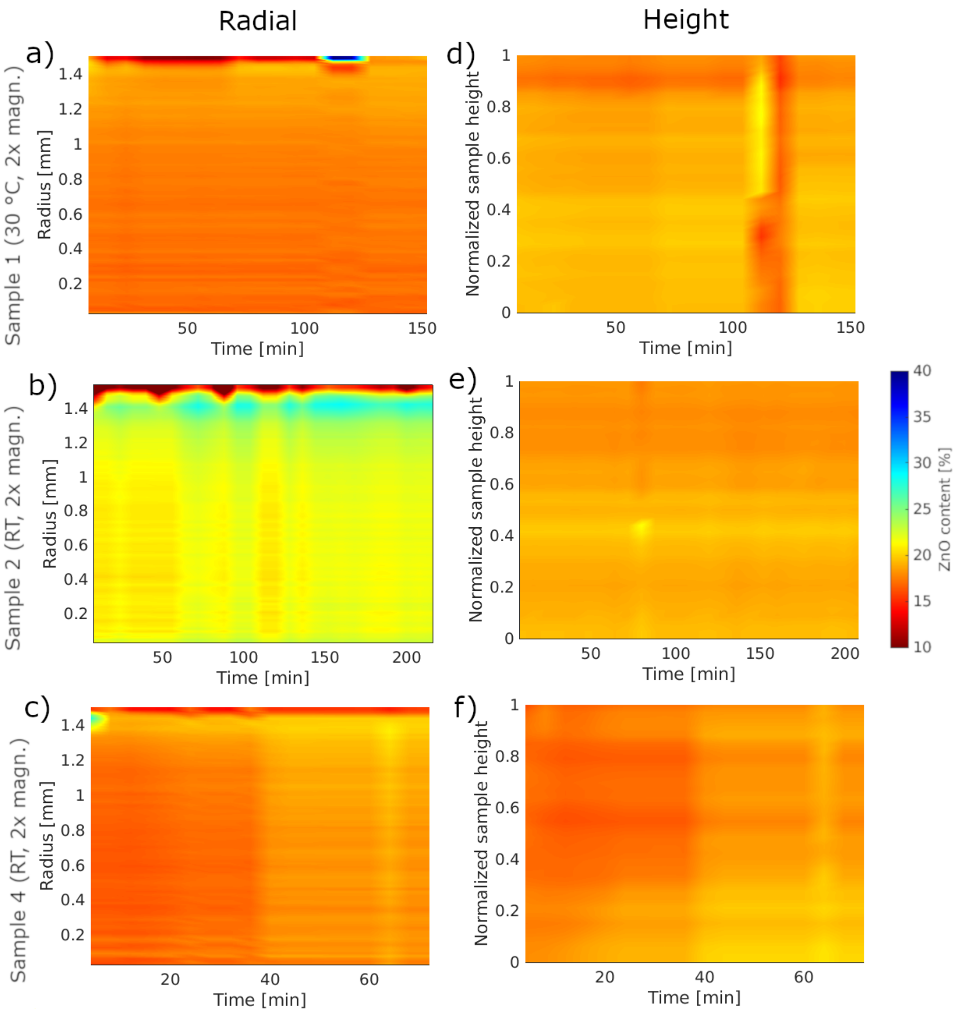

Figure A4: Left column (a-c) shows the radial distribution of t-ZnO in the three samples 1, 2, and 4, while the right column (d-f) shows the height distribution of t-ZnO in the three samples. In theory, the t-ZnO content over time should be constant, which is the case with only small deviations. These deviations are due to some small differences in the segmentation and can be neglected. Similar to the radial water distribution, a slightly lower t-ZnO content is detected at the edges of each sample.

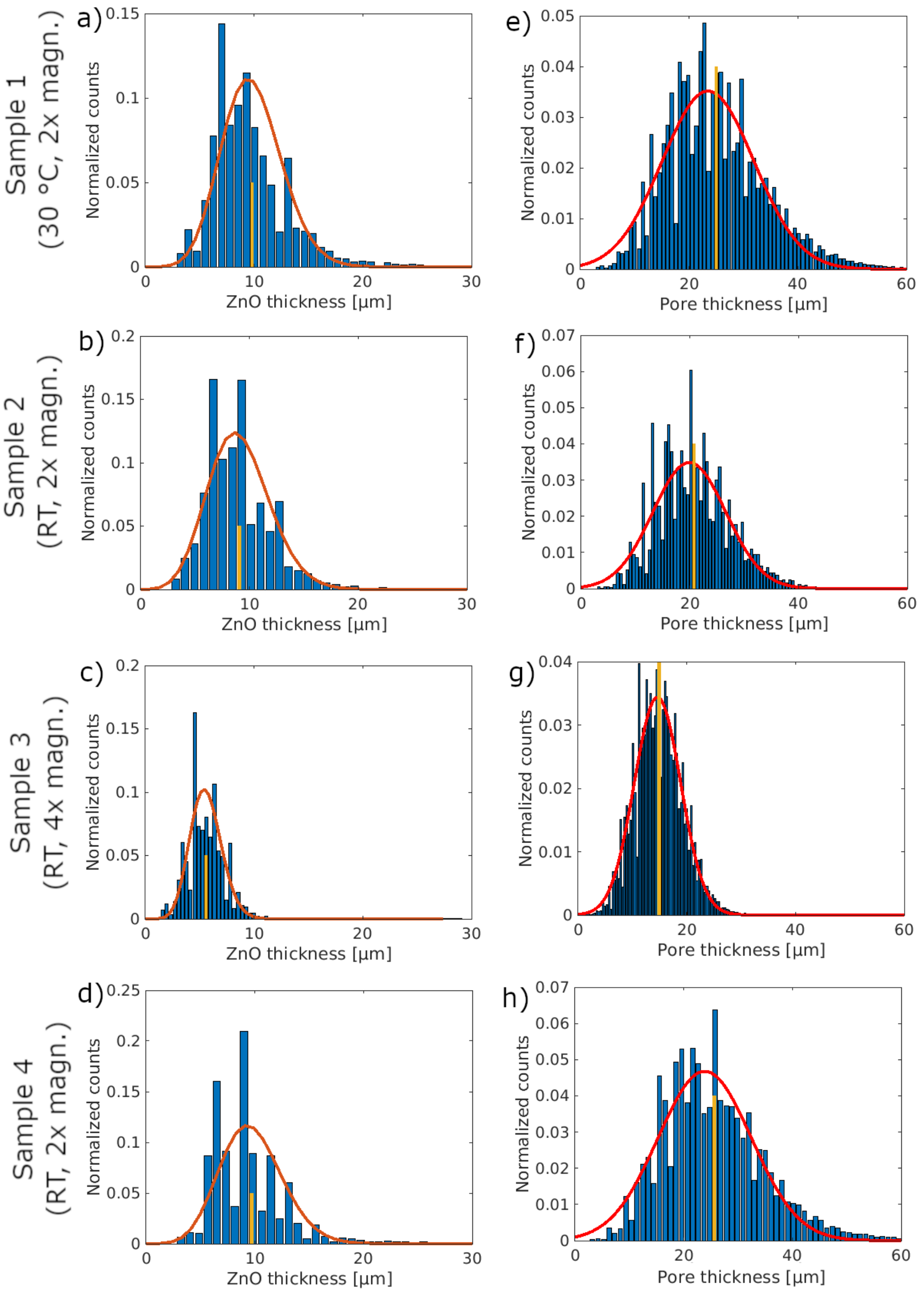

Figure A5: The first row (a-d) shows the t-ZnO arm thickness distributions of the samples 1, 2, 3, and 4, while the second row (e-h) shows the pore size distributions of these samples. The red curves represent the fit distributions, while the yellow bars indicate the weighted mean value of each distribution.
